# Exploring the facilitators and barriers to high-risk behaviors among school transportation drivers: a qualitative study

**DOI:** 10.1186/s12889-022-13630-x

**Published:** 2022-06-23

**Authors:** Shadi Fathizadeh, Mahmood Karimy, Mahmoud Tavousi, Fereshteh Zamani-Alavijeh

**Affiliations:** 1 Student Research Committee, School of Health, Isfahan University of Medical Sciences, Isfahan, Iran; 2 Social Determinants of Health Research Center, Saveh University of Medical Sciences, Saveh, Iran; 3 Health Metrics Research Center, Iranian Institute for Health Sciences Research, ACECR, Tehran, Iran; 4 Department of Health Education and Promotion, School of Health, Isfahan University of Medical Sciences, Isfahan, Iran

**Keywords:** High-risk behaviors, Drivers, School transportation, Safety, Facilitators, Barriers

## Abstract

**Background:**

School transportation (ST) crashes are associated with serious adverse consequences, particularly for students in developing countries. High-risk behaviors (HRBs) of ST drivers are a major factor contributing to ST crashes. This study aimed at exploring the facilitators and barriers to HRBs among ST drivers.

**Methods:**

This qualitative study was conducted in 2019–2020. Participants were ST drivers, students, parents, and school staff purposively selected from Tehran, Iran. Data were collected through in-depth semi-structured interviews and focus group discussions and were concurrently analyzed through conventional content analysis.

**Findings:**

Participants were fifteen ST drivers with a mean age of 45 ± 10.2 years and 24 students, parents, and school staff with a mean age of 28.62 ± 16.08 years. The facilitators and barriers to HRBs came into five main categories, namely previous experiences of HRBs, perceived gains and risks of HRBs, motivating and inhibiting feelings and emotions, positive and negative subjective norms, and perceived mastery in driving.

**Conclusion:**

A wide range of facilitators and barriers can affect HRBs among ST drivers. Strategies for preventing HRBs among ST drivers should be multidimensional and individualized and should focus on strengthening the barriers and removing the facilitators to HRBs.

## Background

School transportation (ST)is an important type of transportation [1]. Some students use ST due to their parents’ employment or their long distance to school [2]. Each day, more than 25 million students in the United States use ST to go to school and return to home [3]. In 2018, around 1.7 million students in Iran used ST [4]. Parents expect their children to go to school and return to home in safety [5] and ST can be an appropriate route for safe student transportation [6]. Nonetheless, ST carries different risks for students, increases their vulnerability [1], and creates heavy socioeconomic burden [7]. Therefore, ST drivers need to prioritize student safety and health [8].

ST crashes in all countries cause serious physical injuries and even death for students and have negative effects on communities [9]. For example, more than forty children in China died during one year due to ST crashes [10]. In the United States, 800 children die each year due to motor vehicle accidents during school time and 2% of these deaths are due to school vehicle accidents [3]. In developing countries, injuries due to ST crashes are more serious and have increasing prevalence [10]. For example, number of student death in ST crashes in Tehran, the capital of Iran, increased from fifteen in 2016 to 22 in 2018 [11]. The most prevalent injuries caused by ST crashes among children less than ten years and children aged 10–19 years are head trauma and lower extremity injuries, respectively [12]. Therefore, ST crashes are considered as serious threats to student health [13]. Safe driving and protecting passengers against potential risks are among the main responsibilities of ST drivers and ST authorities [2]. Nonetheless, drivers’ behaviors are a major factor contributing to traffic accidents [1]. Two studies reported human errors as the main reason of 75%–90% of traffic accidents [14, 15]. ST drivers’ HRBs not only cause accident injuries, but also can negatively affect students’ behaviors [8]. ST drivers are the first and the last individuals who are in contact with students in the time interval between home leaving and returning to home and play significant role in ensuring student safety [2]. However, they may endanger student safety through engagement in HRBs and commitment of driving offenses such as speeding, non-observance of the right of way, carrying excessive passengers, and driving a defective car [3, 9]. Given the high prevalence of ST crashes in Iran [16], the importance of protecting students’ physical and mental health [2]. The significant role of ST drivers in protecting student health [5], and the significant effects of ST drivers’ behaviors on ST crashes [8] and on student behaviors [8], quality education about safe driving and safe ST for ST drivers is necessary to improve their driving behaviors [2, 17]. A key step to educational programs for ST drivers is to study their driving behaviors and their contributing factors. A study in Great Britain reported that the most important factors contributing to traffic accidents among young drivers were risk taking, inexperience, and distraction due to using mobile phone, while the most important factors contributing to traffic accidents among elder drivers were medical conditions, defective eyesight, and slow driver reactions [18]. Other studies also reported driver-related factors, such as recognition and decision errors [19], socioeconomic background [20], fatigue, driving stress, irritability due to long-term driving [21], physical and mental abilities, and personality traits [20], as the most important factors contributing to HRBs among drivers. The contributing factors of HRBs among drivers largely depend on the immediate sociocultural context [20] and hence, the results of studies in this area in one context may not easily be generalizable to other contexts [22, 23]. Some scholars also noted that some contributing factors of HRBs are still unknown [22]. Moreover, there are limited data in this area in Iran [24]. These gaps highlight the necessity of further studies to produce clearer evidence in this area. Therefore, the present study was conducted using a qualitative design in order to explore the facilitators and barriers to HRBs among ST drivers. Social behaviors, such as driving behaviors, are complex phenomena [25]. Scholars believe that quantitative designs are not appropriate for studying complex and poorly known phenomena [22, 25]. On the other hand, qualitative studies are appropriate for exploring complex phenomena, such as driving behaviors, based on the immediate sociocultural factors [22, 26]. Therefore, a qualitative design was used in the present study.

## Methods

### Design

This qualitative study was conducted from April 2019 to March 2020 using conventional content analysis. Conventional content analysis is appropriate for describing poorly known phenomena, about which there are limited theories or literature [27].

### Participants and setting

The main study participants were fifteen male and female ST drivers with rich experience of ST driving in Tehran, Iran. The mean of their age was 45 years. Besides, nine students with a mean age of eleven years, seven students’ mothers with a mean age of 31 years, five students’ fathers with a mean age of 46 years, and three school staff (two school principals and a teacher) with a mean age of 41 years were included in the study in order to explore the different aspects of the facilitators and barriers to HRBs among ST drivers. Sampling was purposively performed with maximum variation respecting the educational degree of students and the geographical area of schools. Participants were selected from all five main geographical areas of Tehran, namely the north, east, west, south, and center of the city.

### Data collection

Data were collected through in-depth semi-structured interviews and focus group discussions started using questions about demographic and occupational characteristics such as age, gender, educational level, main occupation, work experience as ST driver, number of ST services per day, and type of car. Then, broad questions were used to guide the interviews. Examples of these questions for ST drivers were, “Can you describe one of your working days?” and “What factors contribute to your HRBs?” The type of the interview questions for other participants varied according to the gaps in the data. An example was, “Can you explain your experiences of ST driver’s behaviors during ST?” Probing questions such as “Can you explain this more?” “What do you mean?” “Why and how?” and “Can you provide an example?” were also used to further explore participants’ experiences. Participants had the opportunity to freely explain their experiences. The first author and a trained male colleague collected the data in Persian in a safe and quiet place in school dean offices, taxi agency offices, or city streets. Interviews and group discussions lasted 25–40 min, audio-recorded with participants’ permission, and continued up to data saturation, i.e., when no new data were obtained. Accordingly, three focus group discussions with nineteen participants and twenty interviews with twenty participants were held.

### Data analysis

Data were analyzed using the three-step conventional content analysis proposed by Elo and Kyngäs [26]. In the data preparation step, each interview was transcribed word by word and its transcript was perused for several times in order to obtain a general understanding about its main ideas. In the data organization step, the data were reduced through reviewing the transcript and determining and labeling meaning units to generate primary codes. Cods were constantly compared with each other and grouped into subcategories according to their similarities. Similarly, subcategories were compared and grouped into larger categories. Codes, subcategories, and categories were further developed and revised based on new interviews. Finally, the data were reported in the data reporting step.

### Rigor

The trustworthiness of the data was ensured using Lincoln and Guba’s criteria [28], namely credibility, confirmability, and transferability. Credibility was ensured via prolonged engagement with participants for more than one year in order to better understand their experiences. Moreover, data collection and analysis were performed concurrently and circularly. Triangulation of data source and data collection methods was also used to overcome the weaknesses of the different data sources and data collection methods. Constant comparison analysis was also used during data analysis. Confirmability was maintained through member checking by participants and peer checking by coauthors and then, findings were revised according to their comments. Moreover, findings were compared with the findings of previous studies in the external report check process. To ensure transferability, clear descriptions were provided about participants’ characteristics and original data were kept for subsequent assessment. Moreover, the processes of data collection and analysis were described step by step in order to provide others with the opportunity of the stepwise replication of the study.

This study was approved by the Ethics Committee of Isfahan University of Medical Sciences (approval code: IR.MUI.REC.1398.385) and all methods were performed in accordance with the relevant guidelines and regulations.

## Results

Participants were fifteen ST drivers and 24 students, parents, and school staff. ST drivers were ten males and five females with a mean age of 45 ± 10.2 years and a mean work experience of 6 ± 2.96 years. Other participants were eleven students, ten parents, and three school staff (eleven males and thirteen females) with a mean age of 28.62 ± 16.08 years. Table 1 shows participants’ characteristics.

**Table 1:** Participants’ characteristics

Characteristics		STDs	Other participants
		**N (%)**	**N (%)**
Age (Years)	< 15	0 (0)	9 (37.5)
	15–25	0 (0)	2 (8.33)
	26–36	1 (6.6)	3 (12.5)
	36–46	6 (40)	4 (16.66)
	> 46	8 (53.3)	6 (25)
	Mean	45 ± 10.2	28.62 ± 16.08
Gender	Male	10(66)	11(45.84)
	Female	5(33.3)	13(54.16)
Educational level	Below diploma	1 (6.7)	11 (45.83)
	Diploma	9(60	6(25)
	Associate diploma	3(20)	4(16.66)
	Bachelor’s	1(6.7)	2(8.33)
	Master’s and higher	1(6.7)	1(4.16)
Occupation other than STD	Taxi driver	7(46.7)	-
	Employee	1(6.7)	6(25)
	Retired	1(6.7)	-
	Housewife	4(26.7 (	3(12.5)
	Self-employed	2(13.3)	4(16.66)
	Student	---	11(45.83)
Number of daily ST services	1–3	7(46.7)	—
	3–5	5(33.3)	
	> 5	3(20)	
Car type	Sedan	14(93.3)	—
	Van	1(2.7)	
Work experience as STD (Years)	1–5	8 (83.3)	—
	> 5	7 (46.6)	
	Mean	6 ± 2.96	

Data analysis revealed that five main categories of factors can affect ST drivers’ HRBs. These five categories were previous experiences of HRBs, perceived gains and risks of HRBs, motivating and inhibiting feelings and emotions, positive and negative subjective norms, and perceived mastery in driving. The final pattern in the data revealed that each of these factors was a spectrum with facilitators at one end and barriers at the other end (Table 2).

**Table 2:**
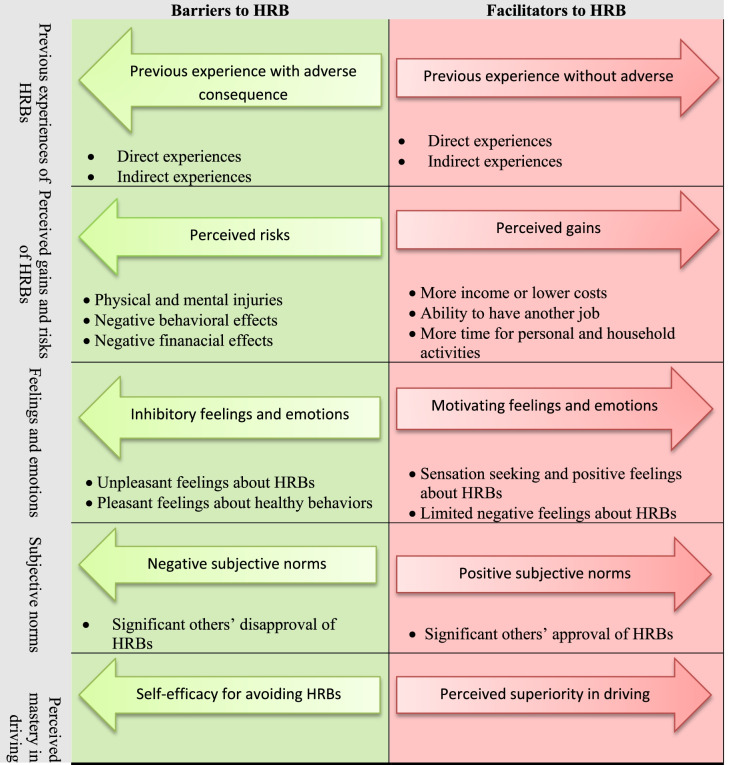
Facilitators and barriers to HRBs

### Theme 1: previous experiences of HRBs

Participants’ experiences showed that previous experiences of HRBs can act on a spectrum as both facilitators and barriers to HRBs among ST drivers. The direct or indirect experiences of HRBs without any adverse consequence at one end of the spectrum were a facilitator to HRBs, while the direct or indirect experiences of HRBs with adverse consequences were a barrier to HRBs among ST drivers.

#### Subthem1: experiences of HRBs without any adverse consequence: a facilitator to HRBs

Some participants reported that their direct experiences of HRBs with no adverse consequence for themselves and others were a facilitator to their HRBs.

*“I have picked up four students on the back seat so far and haven’t experienced any problem. Previously, my car had no seat belt and nothing occurred for my passengers. Therefore, I don’t insist that students should fasten seat belt”* [male ST driver, P1]

Some participating ST drivers also reported that they engaged in HRBs due to witnessing or hearing about the HRBs of their colleagues which had had no adverse consequences.

*“My colleagues always drive the wrong way in this one-way street and have never experienced any problem. I also learned to do so and haven’t experienced any problem so far”* [male ST driver, P5]

*“Most of the times, I used to go inside the alley, ring the doorbell of students’ homes, and delivered students to their parents. However, my colleagues said that they didn’t do so and hence, I changed my habit. Now, I drop off students and leave there”* [male ST driver, P1]

### *Subthem2:* experiences of HRBs with adverse consequences: a barrier to HRBs

Some participating ST drivers highlighted that direct or indirect experiences of HRBs with adverse consequences prevented them from re-engagement in such behaviors.

*“I never pick up extra passengers because sometimes I received heavy penalties or lost my concentration during driving. Last year, I had seven students in my car. They had so serious conflicts with each other that I couldn’t control them”* [male ST driver, P3]

*“One time, the driver sharply braked and I hit to the front seat by the nose and experienced nosebleed. Since then, the driver first gets ensured that all of us have fastened seat belt and then, starts driving*” [female student, P22]

Some participating ST drivers also referred to the adverse consequences of their colleagues’ HRBs as a barrier to their HRBs.

*“I saw that the student got out of the car and closed the door while one end of his bag was still in the car. The driver drove without noticing this and pulled the student with himself several meters. Thereafter, I always get out of the car and deliver students to their parents and leave”* [male ST driver, P6]

### Theme 2: perceived gains and risks of HRBs

According to the participants, perceived benefits and risks of HRBs can affect ST drivers’ HRBs. Perceived gains of HRBs at one end of the spectrum can move ST drivers towards engagement in HRBs, while perceived risks of HRBs at the other end of the spectrum can act as a barrier to HRBs. Gain and risk perceptions are in turn affected by ST drivers’ needs and previous experiences.

#### Subtheme 1: perceived gains: a facilitator to HRBs

The perceived gains of HRBs motivated ST drivers to engage in HRBs. Some participating ST drivers reported that they engaged in HRBs such as speeding, dangerous overtaking, picking up excessive number of passengers, wrong-way driving in one-way streets, and dangerous turning to avoid traffic congestion, shorten their way, transfer students more rapidly, and thereby, earn more income.

*“The more the students I pick up, the higher the income I will have. Sometimes, I drive the wrong way in one-way streets and drive faster to be able to have one more ST service in another school”* [female ST driver, P2]

Some ST drivers also noted that they picked up or drop off students at the top of streets or alleys instead of their home doors and committed some driving offences in order to reduce their gas consumption and car depreciation and save money.

*“Most alleys are narrow and hence, I avoid entering them because turning in narrow alleys may result in damages to my car. Collision repair costs are more than my income. If I want to avoid turning in narrow alleys, I should drive longer distances and pay more for gas”* [male ST driver, P8]

Some ST drivers had rental cars and hence engaged in HRBs such as using shorter paths and wrong-way driving in one-way streets in order to return their rental cars to their owners.

*“I don’t have a private car and rent a van for ST services. I have to use the most of the van when it is in my hands. Therefore, I have to engage in speeding, dangerous overtaking, and dangerous turning”* [male ST driver*,* P10]

Another gain of HRBs was the opportunity to have another job. Most participating ST drivers had another job and considered ST as their second job.

*“I admit that speeding is dangerous; but it helps me have enough time to do all of my activities. Speeding enables me to transport the students of both schools and return to my main job”* [male ST driver, P1]

Moreover, HRBs helped ST drivers have enough time to perform their personal and household activities. Some ST drivers reported that HRBs and violation of traffic rules helped them shorten ST time and save time for their other activities.

*“Sometimes, I have to use shorter paths, engage in speeding, and drive the wrong way in one-way streets in order to have more time for my children and household activities”* [female ST driver, P2]

### *Subtheme 2: perceived risks*: a barrier to HRBs

The perceived risks of HRBs were a major barrier to HRBs. One of these risks was injuries to students due to HRBs.

*“I don’t know how some drivers dare to drop off students in street. I don’t dare because they are at risk for accidents”* [female ST driver, P9]

Most participants agreed that HRBs, such as dropping off students in unsafe places, no predetermined time for ST, and change of car driver without previous announcement, not only can cause physical injuries, but also can negatively affect the mental health of students and families.

*“I always pick them up right at the predetermined time. Missing a student causes the student stress and causes families distrust and upset”* [male ST driver, P3]

*“My son had been left behind the door without being able to ring the doorbell. In these cases, my little son is at risk for different adverse events. What if they kidnap my son? Drivers will never leave a child alone if they perceive these risks”* [student’s mother*,* P30]

Some participants also referred to the negative educational and behavioral effects of HRBs as a barrier to HRBs. They noted that any HRB or rule violation can negatively affect students’ mentality and behaviors.

*“ST drivers should be good role models for students. Unfortunately, some ST drivers don’t have appropriate behaviors. Students spend about one hour of their time each day with ST drivers and hence, ST drivers’ violation of rules can waste parents’ and teachers’ attempt for educating students”* [male school staff, P38]

Some ST drivers also reported financial disadvantages of HRBs such as damage to car and suspension of their job as a barrier to their HRBs and noted that the financial consequences of HRBs may be far beyond ST drivers ’ financial affordance.

*“Using a mobile phone seriously distracts me. It may result in damages which affect a driver for life. HRBs are not worthy of endangering students’ lives”* [male ST driver, P6]

### Theme 3: motivating and inhibiting feelings and emotions

Participants’ experiences showed that feelings and emotions can motivate or inhibit engagement in HRBs. Sensation seeking, pleasant feelings, and management of negative feelings were facilitators to HRBs, while unpleasant feelings about HRBs were a barrier to HRBs.

#### Subtheme 1: motivating feelings and emotions: a facilitator to HRBs

Some participating ST drivers reported engagement in HRBs and violation of traffic rules to seek sensation and pleasant feelings.

*“Speeding, weaving through the traffic, and tailgating are exciting to me because students in car greatly encourage me with pleasure”* [male ST driver, P8]

Moreover, negative feelings such as fatigue and low mood can move ST drivers towards the violation of traffic rules.

*“I felt tired and was not in mood to stop behind the street light. Thus, Iran the red light”* [male ST driver, P7]

#### *Subtheme 2: inhibiting feelings and emotions*: a barrier to HRBs

Participants’ experiences showed that some ST drivers felt tension, unpleasant feelings, and pangs of conscience during HRBs and had good feelings and satisfaction when they could have healthy behaviors and observe traffic rules.

*“Last year, I picked up six and sometime seven students. I didn’t want to do so but our contractor required us to do so”* [female ST driver, P4]

### Theme 4: subjective norms

Subjective norms or others’ opinions about HRBs were also among the facilitators and barriers to HRBs. Participants’ experiences showed that others’ approval of HRBs facilitated their engagement in HRBs, while others’ disapproval of HRBs was a barrier to HRBs.

#### Subtheme 1: significant others’ approval of HRBs: a facilitator to HRBs

Some participating ST drivers noted that they highly valued their friends’ and colleagues’ opinions about their driving and reported engagement in HRBs with their friends’ and colleagues’ approval.

*“It’s for several years that I’m a driver and interact with many drivers. My colleagues’ opinions are important to me. For example, they disapprove picking up students at their home door”* [male ST driver, P1]

#### *Subtheme 2: significant others’ disapproval of HRBs*: a barrier to HRBs

Participants’ experiences showed that the disapproval of HRBs by significant others including family members, students, police, and school staff made ST drivers avoid HRBs.

*“Once, I was in hurry and wanted to run the red light. Students complained and thereby, made me not run the red light. Most of the times, they prevent me from violating traffic rules”* [male ST driver, P6]

### Theme 5: perceived mastery in driving

Participants reported perceived mastery in driving, perceived ability to engage in HRBs, and perceived ability to avoid HRBs as the facilitators and barriers to HRBs. The two subcategories of this category were perceived superiority and self-efficacy for avoiding HRBs.

#### Subtheme 1: perceived superiority: a facilitator to HRBs

Perceived superiority in driving was a major facilitator to HRBs. Participants’ experiences showed that some ST drivers felt more experienced and more competent than other drivers and believed that they had mastery in driving and hence, engaged in HRBs. A young ST driver with limited driving experience explained his competence in driving by saying,

*“I have excellent car handling skills. I can brake and stop as needed. I’m superior in weaving through the traffic. It is impossible that I make any mistake while driving”* [male ST driver, P10]

#### *Subtheme 2: self-efficacy for avoiding HRBs*: a barrier to HRBs

Some participating ST drivers reported that ST drivers can avoid HRBs if they believe in their ability to engage in healthy behaviors, avoid risky situations, and observe traffic rules.

*“I can take students healthy to school or their homes without committing any driving offence which can endanger my life or students’ lives”* [female ST driver, P12]

## Discussion

This study aimed at exploring the facilitators and barriers to HRBs among ST drivers. Findings revealed that the major facilitators and barriers to HRBs among ST drivers were previous experiences of HRBs, perceived gains and risks of HRBs, motivating and inhibiting feelings and emotions, positive and negative subjective norms, and perceived mastery in driving. These barriers and facilitators are discussed in what follows.

### Theme 1: previous experiences of HRBs

Findings showed that previous direct and indirect experiences of HRBs with or without negative consequences acted on a spectrum as facilitator and barrier to HRBs among ST drivers. In line with this finding, previous studies in China [29], Cyprus [30], and Spain [31] also reported that previous experiences of traffic accidents increase risk perception and thereby, act as a barrier to HRBs and a facilitator to engagement in protective behaviors [32]. However, a study reported that previous experiences and risk perception may not necessarily lead to protective behaviors among ST drivers [32]. Another study in South Africa also showed that accidents had no significant effects on risk taking among taxi drivers [33]. It seems that the consequences of previous HRB-related experiences may not have strong inhibitory effects to prevent ST drivers’ re-engagement in HRBs. The findings of the present study respecting the effects of previous experiences of HRBs can be used to redefine the concept of “previous experiences” in the Self-Efficacy Theory [34]and the Social Cognitive Theory [35]. Moreover, our findings highlight the need for developing more effective road safety programs to reduce HRBs among drivers who frequently engage in them [21]. The developers of educational programs can use messages about the negative HRB-related experiences of drivers and the negative consequences of HRBs (such as physical disability and financial problems) in order to correct other drivers misconceptions about HRBs.

### Theme 2: perceived gains and risks of HRBs

Our findings also showed that ST drivers’ perceptions of the gains and the risks of HRBs can act as a facilitator or a barrier to HRBs. Similarly, the Prospect Theory holds that weighing advantages of a behavior against its disadvantages affects engagement in that behavior [36]. One of the reasons of ST drivers in the present study for engagement in HRBs was personal or familial gains such as the possibility to earn more income. This is in line with the findings of two former studies which reported perceived benefits as an influential factor in modifying health-related behaviors [37, 38]. Pender also highlights that individuals usually select behaviors which are most beneficial [39]. Moreover, a study showed that perceived benefits of HRBs require drivers to engage in HRBs [40]. Two other studies also found that HRB benefits such as early arrival at destination, perceived superiority over other drivers, ability to concurrently perform several tasks [40, 41], saving more time, and sense of freedom [42] were among the facilitators of drivers’ engagement in HRBs. A study on drivers in Australia also reported the better use of time as a benefit of using mobile phone while driving [43]. Moreover, our findings revealed that some ST drivers engaged in HRBs in order to be able to have more time for their other job(s). Great fatigue due to having two or more jobs can impair concentration and functioning, cause frequent distractions, increase the likelihood of engagement in HRBs, and increase the risk of accidents.

On the other hand, study findings showed that perceived risks of HRBs, such as physical and mental injuries and financial problems, acted as a barrier to HRBs. Perceived risks can affect behavioral intention [44] and behavior [45] so that personal differences in risk perception can explain differences in engagement in HRBs such as traffic rule violation [46]. A study in Australia showed that higher perception of the risks of unsafe driving is associated with lower probability of engagement in HRBs and violation of traffic rules, though some drivers may engage in HRBs despite knowing their risks and disadvantages [43]. Individuals weigh the gains of a given behavior against its risks and then, decide to engage or not to engage in that behavior [47]. HRBs can cause adverse consequences for different people [48]; nonetheless, individuals may decide to engage in them based on their perceptions of the potential gains or risks. Therefore, simple strategies, such as risk messages, which focus on improving individuals’ understanding of HRB-associated risks may not be effective enough to motivate ST drivers to avoid HRBs. Comprehensive educational interventions to highlight the importance of the risks of HRBs and the unimportance of HRB-associated gains may help drivers decide not to engage in them.

### Theme 3: motivating and inhibiting feelings and emotions

Study findings also indicated that feelings and emotions can affect STdrivers’ HRBs. In line with the findings of two former studies [49, 50], our findings revealed that negative feelings such as fatigue and low mood can facilitate ST drivers’ engagement in HRBs. Moreover, we found sensation seeking as a facilitator to HRBs. Similarly, two studies reported that drivers who enjoy HRBs are more likely to engage in them [51, 52]. Sensation seeking has significant role in determining driving behaviors and driving culture and significantly increases accident-related injuries [53]. High levels of sensation seeking may be associated with higher probability of engagement in HRBs such as speeding, not fastening seat belt, drunk driving, and competition with other drivers [54]. On the other hand, our findings showed tension, unpleasant feelings, and pangs of conscience after HRBs as barriers to HRBs. The Cognitive Dissonance Theory [55] also holds that behaviors which are incongruent with individuals’ cognitions cause them tension and unpleasant feelings and hence, they attempt to avoid such behaviors in order to prevent such feelings and modify their behaviors to have pleasant feelings [55, 56]. Previous studies showed that appropriate educational interventions can be used for attitude and behavior modifications and promote healthy behaviors among individuals with HRBs [57, 58].

### Theme 4: positive and negative subjective norms

Study findings showed that positive and negative subjective norms can affect ST drivers’ HRBs. The Theory of Planned Behavior also states that perceived pressure by significant others can affect engagement in a given behavior [59]. Two other studies also reported that significant others’ pressure has significant effects on behaviors [60, 61]. Our findings also revealed that positive subjective norms were a facilitator to HRBs. This is in agreement with the findings of two previous studies in Iran [20, 62]. On the other hand, our findings revealed that negative subjective norms, such as the negative attitudes of families, parents, school staff, and police, acted as a barrier to HRBs. Similarly, two former studies reported the significant effects of subjective norms on HRBs among drivers [60, 63]. These findings highlight that colleagues’ and significant others’ negative attitudes towards HRBs can reduce the prevalence of HRBs among ST drivers. Therefore, safety-based educational interventions for students, parents, and drivers can reduce HRBs among drivers.

### Theme 5: perceived mastery in driving

We also found that perceived mastery in driving acted as a facilitator and a barrier to HRBs among ST drivers so that perceived superiority in driving moved ST drivers, particularly the younger ones, toward engagement in HRBs. In agreement with this finding, a previous study found that drivers who overestimated their driving mastery authorized themselves for engagement in HRBs [64]. Overestimation of driving mastery and low risk perception can make drivers violate traffic rules and engage in HRBs, particularly speeding [30]. On the other hand, perceived self-efficacy for avoiding HRBs was found in the present study as a barrier to HRBs among ST drivers. Self-efficacy refers to individuals’ perceptions of their control over their behaviors [65] or their perceived ability to avoid risky or unhealthy behaviors [59]. Self-efficacy is a significant predictor of behavioral intention and safe behavior, particularly with respect to speeding [65]. A study in Spain also reported self-efficacy as a significant determinant of drunk driving [66]. Compared with other factors, personal factors have the greatest effects on drivers’ engagement in HRBs and hence, educational interventions are essential to modify drivers’ beliefs and perceptions. Educational messages about the consequences of HRBs can be used to improve drivers’ risk perception and thereby, reduce their engagement in HRBs.

This study had three main limitations. First, some ST drivers refused participation in the study due to their concerns over losing their job. Second, like all qualitative studies, this study was conducted on a small sample of individuals and hence, findings may have limited generalizability. Third, as most ST drivers in Iran are male, most study participants were male ST drivers and we could not compare the HRB-related experiences of male and female ST drivers. A strength of the study was the inclusion of individuals with a wide range of direct and indirect HRB-related experiences. Moreover, the present study provided a basis for further studies into HRBs of ST drivers in psychological or behavioral paradigms.

## Conclusion

This study suggests that previous experiences of HRBs, perceived gains and risks of HRBs, feelings and emotions, positive and negative subjective norms, and perceived mastery in driving can act as facilitators and barriers to HRBs among ST drivers. Moreover, this study highlights that ST drivers’ engagement in HRBs largely depends on their HRB-related beliefs, perceptions, and experiences. ST drivers with greater risk perception and firmer belief in the negative consequences of HRBs are more likely to avoid these behaviors. On the other hand, the significant contribution of the perceived gains of HRBs to ST drivers’ engagement in HRBs highlights the need for modifying ST drivers’ perceptions about the triviality of the gains in comparison with the risks of HRBs. Moreover, this study shows that despite good risk perception, some ST drivers may still engage in HRBs due to their perceived superiority in managing potential HRB-related risks. Perceived superiority is a poorly known factor in the area of HRBs among ST drivers and deserves further exploration. Given the wide variety of the facilitators and the barriers to HRBs among ST drivers, one-size-fits-all approaches cannot be used to prevent ST drivers’ HRBs. Rather, individualized approaches should be developed based on the characteristics of each ST driver in order to more effectively prevent HRBs and their associated negative physical, mental, and behavioral consequences.

## Data Availability

The datasets generated during and analyzed during the current study are not publicly available due to [the regulations of the Research Committee of Isfahan University of Medical Sciences, ethical sensitivity and the sensitive nature of interviews transcript data, which are including risky behaviors performed by drivers. Publication of entire transcripts risk identifying research participants. But are available from the corresponding author on reasonable request.

## Abbreviations

ST: School transportation
ST driver: School transportation driver
HRB: High-risk behavior
